# Evaluating the results of long tubular bone distraction with an advanced rod monolateral external fixator for achondroplasia

**DOI:** 10.1038/s41598-021-94146-z

**Published:** 2021-07-19

**Authors:** Bolatbek Dossanov, Vitaliy Trofimchuk, Vassiliy Lozovoy, Sergey Khmyzov, Assem Dossanova, Olzhas Zhukenov, Nazym Tuktiyeva, Aleksandr Angelov

**Affiliations:** 1 Department of Pediatrician Surgery, NSC Astana Medical University, Nur-Sultan, Kazakhstan; 2 Department of Pathology of the Spine and Joints of Children, Sitenko Institute of Spine and Joint Pathology, Kharkiv, Ukraine; 3 Department of Pediatrician Surgery and Orthopedic, NSC Semey Medical University, Semey, Kazakhstan

**Keywords:** Health care, Medical research

## Abstract

The work aimed to evaluate the effectiveness of the developed distraction system based on the rod external monolateral fixation mechanisms by comparing it with the classical technique of long tubular bones distraction based on the circular multi-axial system. The study included patients with a genetically confirmed diagnosis of achondroplasia. The experimental group consisted of 14 patients who underwent surgical limb lengthening by the rod monolateral external fixator with a distraction system developed by the authors. The lengthening was performed on 28 segments of tubular bones. The majority of the experimental group patients achieved the lengthening value close to the planned one and the deformation correction. The fixation period was averagely 83.8 ± 3.7 days, the regenerate length was 8.5 ± 0.6 cm, and the mechanical strength of the distraction regenerate was 10.3° ± 2.18°. The rod external fixator with a control distraction system developed by the authors has small dimensions and low weight of the external supporting elements of high durability. It is reported to provide a good psychological tolerance of the treatment process and significantly outperforms the circular multi-axis system. Considering the aforementioned, the proposed apparatus can grant good orthopedic care to patients with achondroplasia.

## Introduction

Achondroplasia is defined as a genetically determined systemic disease of the skeleton in children with a predominantly impaired endochondral growth of long and short tubular bones. As a result, there is a pronounced delay in the growth of the limbs, but the spine can reach normal length, which leads to a typical imbalance in body size^1^.

The decision to increase the height in patients diagnosed with this skeletal disorder is specified mostly by the need to lengthen the limbs in order to restore the normal proportions between the torso and limbs, as well as facilitate self-care, for personal and social rehabilitation^2,3^.

To date, the most effective method for correcting the growth deficit in patients with achondroplasia is surgical treatment. The method of distraction osteosynthesis is considered as the main way of limb elongation^4,5^. Lengthening of the lower limbs, as a rule, involves the use of a fixator for the tibia and femur bones, while the upper limbs are lengthened through the bone of the upper arm^6^. More often, external fixators of different designs are employed(monolateral, bilateral, sector, semi-circular, circular, or combined), fundamental studies on processes of reparative tissue regeneration, and the proposed variants of surgical interventions have not lost their relevance up to the present time^7–10^.

In external fixation apparatuses, precision elements can be realized on spokes, rods, or their combinations. Nowadays, more preference is given to the rod monolateral external fixators due to their smaller size and weight, as well as higher convenience for patients^11^. Surgical interventions to correct segment length using rod fixators are almost bloodless, have a minimal risk of damage to the neurovascular bundles during the rods conduction, and cause fewer cases of purulent complications due to greater stability of the rod-bone system.

According to the traditional Ilizarov method, the patient needs to wear an external fixator during both stages, which may increase the pain and the risk of inflammation around the transosseous elements. For this reason, Intramedullary Limb Lengthening methods like the PRECICE system have been developed, assuming a long rod inserted directly into the bone and controlled by powerful magnets from outside^12^. However, the rods used in the Intramedullary Limb Lengthening system are produced from bioinert materials, mainly expensive titanium alloys. Internal fixators do not cause the most of complications compared to external ones, but their application may require a minimum bone diameter. Also, they may break or be in a defective condition^13,14^, which might result in negative consequences as the whole process must be well planned since no postoperative changes (except for the distraction rate) can be made. Besides, some internal fixators, such as PRECICE, cannot be applied on the upper limbs^12^.

Combined methods such as LON (Lengthening Over Nail) and LATN (Lengthening and Then Nailing) can be used as well^15,16^. Their essence consists of removing the external fixator after the distraction period allowing for the consolidation period to proceed only with the internal fixation mechanism. At that, the period of wearing the external fixator reduces in half.

A new tendency of medication limb elongation is also worth mentioning, in particular, the Vosoritide drug and some other establishments. The results of such a method are very encouraging, although this direction is at the stage of clinical trials so far and cannot serve as an alternative to surgical treatment with fixation systems^17–19^.

Therefore, the confirmed efficacy of the Intramedullary Limb Lengthening system and combined methods does not negate the need of developing cost-effective, small-sized, efficient, and patient-friendly external fixators, which can also be employed in combined osteosynthesis treatment techniques.

The authors propose an improved rod monolateral external fixator with an advanced distraction control system. This mechanism is small-sized and convenient for medical and rehabilitation procedures.

The purpose of the work is to evaluate the effectiveness of the developed distraction system based on the rod monolateral external fixation mechanism by comparison with the classical method of long tubular bone distraction at achondroplasia based on the circular multi-axis system.

## Results

The majority of the experimental group patients achieved the lengthening value close to the planned one and deformation correction. At that, the deviation of the achieved value from the planned one is not statistically significant. The results of surgery and information about the experimental group patients are presented in Table 1. The time of fixation in the device was 83.8 ± 3.7 days on average. For the upper arm, it was slightly lower (76 ± 1 days) and for the femur—slightly higher (87.5 ± 2.5 days), although these differences have no statistical significance.

**Table 1:** Results of transosseous osteosynthesis using an advanced rod monolateral external fixator (experimental group).

Patient	Gender	Age	Segment	Consolidation period (days)	Planned lengthening value (cm)	Result of lengthening (cm)	Mechanical stability of the regenerate (degrees)	Deformity angle (degrees). Antecurvation, varus, and valgus	Soft tissue inflammati on (scores)	Pain intensity on the 2^nd^ day after surgery (scores)^a^	Achilotomy Z
1	Boy	5	Lower leg	82	10	8.3	7	Antecurvation 5	0	3	Needle
						8.5		Antecurvation 7	0		
2	Boy	7	Lower leg	85	10	8.9	10	Valgus 4	0	3	Needle
						8.4		Valgus 4	0		
3	Boy	5	Lower leg	85	10	7.9	9	Antecurvation 5	1	3	Needle
						8.2		Antecurvation 3.5	0		
4	Girl	5	Lower leg	79	10	8.3	12	Antecurvation 7.2	0	4	Needle
						8.3		Antecurvation 2	0		
5	Boy	6	Lower leg	80	10	10	12	–	0	3	Needle
						10.2		–	0		
6	Boy	5	Lower leg	88	10	9.1	8	Valgus 1.7	0	3	by Stayer
						8.9		Valgus 1.9	0		
7	Boy	6	Lower leg	87	10	10.3	15	Valgus 1.8	0	3	by Stayer
						9.9		Valgus 1.4	0		
8	Girl	8	Lower leg	85	10	8.2	14	–	0	3	by Stayer
						8.5		–	0		
9	Girl	8	Femur	85	8.5	8.3	9	Varus 7.8	0	3	–
						8.3		Varus 4.8	0		
10	Girl	12	Femur	90	8,5	7.2	7	Varus 5.1	1	3	–
						7.2		Varus 1.9	1		
11	Boy	9	Femur	90	8,5	9	12	Varus 5.1	0	3	–
						9		Varus 5.4	0		
12	Boy	6	Femur	85	8	8	7	Antecurvation 2.2	0	3	–
						8.2		Antecurvation 3.1	0		
13	Girl	15	Upper arm	75	9	7.5	10	–	0	3	–
						7.8		–	0		
14	Girl	10	Upper arm	77	8	8.1	12	–	0	3	–
						8.2		–	0		

^a^0 points in all patients on 18th day after surgery after estimating pain intensity according to Wong-Baker faces pain rating scale.

The length of the regenerate was approximately 8.5 ± 0.6 cm. Length of the upper arm bone before operation made 15 ± 1 cm on average, and after lengthening—22.9 ± 0.76 cm. The average increase in bone length was 53 ± 5%. Length of a lower leg before operation amounted to approximately 16.3 ± 1.4 cm, and after the operation—24.7 ± 1.6 cm. The average gain of the bone length made 52.0 ± 8.2%. The length of the femur before the operation was on the average 28.0 ± 4.6 cm, and after lengthening—36.2 ± 4.1 cm. The average growth of bone length was 30.0 ± 6%. Mechanical stability of the distraction regenerate ranged from 7° to 15°, on average, 10.3 ± 2.18°. The correlation between the bone segment and the length of the regenerate, the bone segment and the mechanical stability of the distraction regenerate is statistically insignificant. No statistically significant correlation between the patients’ age and the above-mentioned parameters was revealed.

Deformities at configuration of bone regenerate were observed on both sides in 6 out of 8 patients at lengthening the bone of the lower leg and in all 4 patients at femur lengthening. At lower leg elongation, antecurvation deformity was observed on 6 bone segments with angles from 2° to 7.2°, varicose deformation was noticed on two segments with angles of 1.4° and 1.8°, and valgus deformation was recorder for two segments with angles from 1.7° to 4°. At femur lengthening, the antecurvation on both limbs with angles 2.2° and 3.1° was noted for 1 patient, and varus deformity on three extremities with angles 1.9° to 7.8° was observed in three patients. Partial or absolute post-distraction shortening of Achilles tendon was recorded for all patients with the lower leg lengthening and eliminated by needle aponeurotomy in 5 patients or achillotomy by Strayer in 3 patients. The pain index in 13 patients on the 2nd day after the surgery was estimated at 3 points according to the Wong-Baker scale, in one patient—at 4 points, and on the 18th day after the operation, the pain index in all patients was zero.

The obtained results showed that at lengthening any segment, the overall nature of the patient's complaints is the same, but the relative importance of particular complications and their severity changes considerably. Almost 60% of all the side effects were joint contractures and soft tissue inflammation. The ankle contracture (equinus) resulted from a shortening of the heel tendon (equinus) due to insufficient joints elaboration and the absence of foot supports. All contractures were eliminated with timely intensification of joint development.

The most frequent complaints from patients and parents were minor inflammation of soft tissue around the rods, which seized after conservative treatment. No cases of necessity to remove the rods and repeat the surgery was registered.

The results of lengthening and information about patients of the control group are presented in Table 2.

**Table 2:** Results of transosseous osteosynthesis using the circular multi-axis system (control group).

Patient	Gender	Age	Segment	Consolidation period (days)	Result of lengthening (cm)^a^	Deformity angle (degrees): Antecurvation, varus, and valgus	Complications	Pain intensity on the 2^nd^ day after surgery (scores)^a^	Pain intensity on the 18^th^ day after surgery (scores)^a^	Achilotomy Z
1	Girl	7	Upper arm	90	7	Antecurvation 18	Spinal osteomyelitis	4	3	–
					7	Antecurvation 17,6	–			
2	Girl	6	Lower leg	92	8	Antecurvation 20	–	3	1	Z
					8	Antecurvation 20				
		7	Femur	105	8	Valgus 13	–	5	2	–
					8	Valgus 17				
3	Girl	9	Femur	107	8	Valgus 20	Intermuscular phlegmon	6	2	–
					8	Antecurvation 16	–			
4	Boy	8	Femur	102	6	Antecurvation 15	Needle fracture	5	2	–
					6	Antecurvation 17	–			
		9	Lower leg	95	6	Valgus 25	Contracture of knee joints	5	2	Z
					6	Valgus 25	Contracture of knee joints			
5	Girl	13	Femur	105	7	Antecurvation 15	–	6	3	–
					7	Antecurvation 15	–			
		14	Lower leg	103	7	Valgus 25	Contracture of knee joints	3	3	Z
					7	Valgus 25	Contracture of knee joints			
6	Girl	14	Lower leg	110	5	Varus 5	–	4	1	Z
					6	Varus 5	–			
7	Boy	7	Femur	107	5	Antecurvation 20	–	3	0	–
					5	Antecurvation 20	–			
8	Boy	6	Lower leg	95	6	Valgus 20	–	3	1	Z
					6	Valgus 20	–			
		7	Femur	107	6	Varus 15	–	4	2	–
					6	Varus 15	–			
9	Girl	6	Lower leg	97	6	Valgus 18	–	2	0	Z
					6.2	Valgus 18	–			
		7	Femur	105	6.5	Antecurvation 17.6	–	5	2	–
					6.5	Antecurvation 17.6	–			

^a^Planned lengthening value is not known; the mechanical stability of the regenerate was not performed.

The planned lengthening value in the control group is not known. The period of fixation in the apparatus was 101.4 ± 5.4 days on average. The regenerate length was approximately 6.6 ± 0.8 cm. Mechanical stability of a distraction regenerate was not defined. Deformities at bone regenerate formation were observed on both sides in all patients of the control group. Antecurvation was noted on 13 bone segments with angles from 15° to 20°, varus deformity—on 4 segments with angles 5° to 15°, and valgus deformity—on 9 segments with angles from 13° to 25°. The shortening of Achilles tendon was observed in all patients with the lower leg lengthening and was eliminated by open Z-achillotomy. The Wong-Baker faces pain rating was 4.1 ± 1.02 on the 2nd day and 1.7 ± 0.8 on the 18th day after the operation.

One patient was diagnosed with spinal osteomyelitis, another suffered from intermuscular phlegmon, and in 4 cases, the knee joint contracture was noticed, which was not fully corrected. One case of a needle fracture was recorded.

## Discussion

From the aforementioned, it can be seen that the fixation time to the full functionality of the regenerate by applying the rod monolateral external fixation apparatus was on average 17% less than by using a circular multi-axis system (Spearman correlation factor rs = 0.765, *p* < 0.05). At the same time, the average lengthening value was 29% higher (rs = −0.545, *p* < 0.05). The information on bone lengthening techniques varies in available scientific sources. Some authors, like Dr. Paley, report that the elongation of the lower limbs up to 40 cm, or more than 50% of bone length in 3 or 4 steps, is possible to perform without subsequent complications^14^. Others argue that no more than 50% of total bone length should be elongated (no more than 10 cm) due to abnormal growth rates after limb lengthening^20,21^. However, to obtain more than 10 cm growth, it is required to undergo more than one treatment. The coefficient of elongation varies to 1 mm per day, although it can be increased in case the process succeeded^6^. Thus, the size of the regenerate achieved in the experimental group can be considered as a good result, and the regeneration rate in both groups was standard for this procedure. The fixation time in the apparatus before the maturation of the distraction regenerate did not exceed that for other types of apparatuses of external fixation when applying the rod external fixator with an advanced distraction system^20,22–24^.

The appearance of different deformities like antecurvation, valgus, and varus, is common phenomena during the bone lengthening, which can be though easily corrected at small angles during this procedure. The deformity angles in forming the bone regenerate of external fixation proved to be significantly smaller in terms of statistics (*p* < 0.01) and, accordingly, easier to correct in the experimental group rather than in the control one. In 2 patients of the control group, the valgus deformity of the lower leg was not fully correlated. Statistically significant results were noted in terms of pain intensity both on the second day after surgery and external fixation (rs = 0.641, *p* < 0.05) and after 18 days (rs = 0.867, *p* < 0.05). It should also be noted that the frequency and severity of complications in the experimental group are lower than those in the control group. Thus, the symptoms of joint contractures in the experimental group patients were prevented by applying intensive gymnastics, whereas in 4 patients of the control group, they remained pronounced. While only slight inflammation of tissues near the rods was observed in several patients of the experimental group, more significant complications, such as spinal osteomyelitis and intermuscular phlegmon, were recorded for the control group.

Side defects occurring during treatment can be related to partial instability of the rods in the bone, which depends on the density of the bone structure at the time of fixation or lack of parental attention at the care of the device^25^. Equinus deformity of the foot in lower leg lengthening was established to be caused by non-adherence to recommendations on wearing and mounting the foot supports in children.

Despite the duration of the treatment, patients noted a significant cosmetic effect, improved gait, better ability of self-care, and higher self-esteem.

## Conclusions

Operative measures on the limb lengthening applying rod external fixator with the elaborated control distraction system refer to a minimally invasive, reliable, and safe surgical methods of achondroplasia treatment in children in comparison with the classical method of long tubular bones distraction based on the circular multi-axis system. Application of this technology in lengthening limb segments allows reaching the stability of the device in the distraction process providing a strong and even regenerate. The best results have been achieved in terms of fixation time, length of the regenerate, deformation angles (antecurvation, valgus, and varus), pain intensity indicators, and complications. The outer supports of the rod external fixator with improved control distraction system are quite small in size and lightweight. Besides, being highly stable, they provide good psychological tolerance of the treatment process and demonstrate a significant advantage over the circular multi-axis system. Taking into account the low cost of described fixation system, it can contribute to the provision of good orthopedic care in achondroplasia patients.

## Materials and methods

### Patients

The experimental group consisted of 14 patients with a genetically confirmed diagnosis of achondroplasia, who underwent surgical limb lengthening using the rod monolateral external fixator within the period from August 2018 to January 2020. Totally, in the experimental group, elongation was performed on 28 segments of tubular bones, namely, on the upper arm—4, thigh—8, and lower leg—16. The average age at the time of surgery was 7.6 ± 2.3 (5–15) years. Distribution by gender was 8 boys and 6 girls.

Control group comprised 9 patients with a genetically confirmed diagnosis of achondroplasia, to whom surgery on limb lengthening was performed with the help of a circular multi-axis system in the period from January 2012 to July 2018. Five patients were treated with tubular elongation of the femur and tibia in two stages. A total of 28 tubular bone segments, particularly, 2 upper arms, 14 femurs, and 12 lower legs, were lengthened in the control group. The average age at the time of surgery was 8.6 ± 2.3 (6–14) years. Distribution by gender was 3 boys and 6 girls.

### Apparatus of external fixation

**Figure 1:**
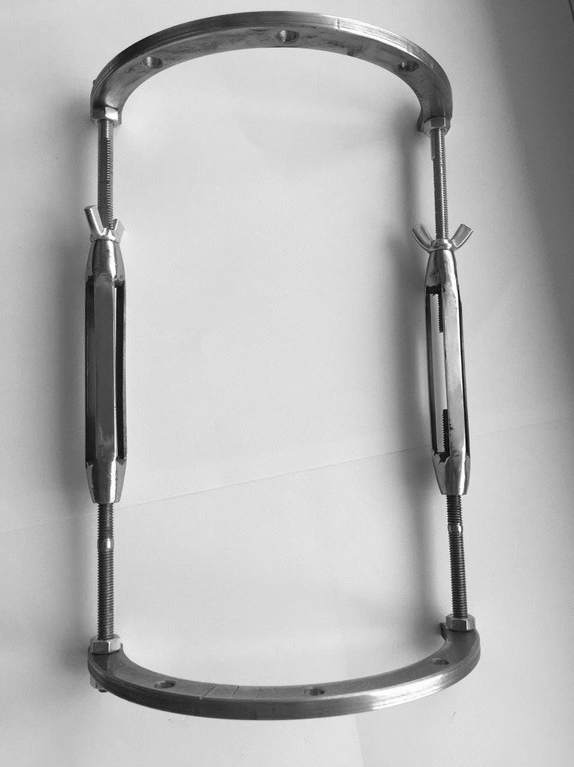
The exterior of advanced rod monolateral apparatus of external fixation.

**Figure 2:**
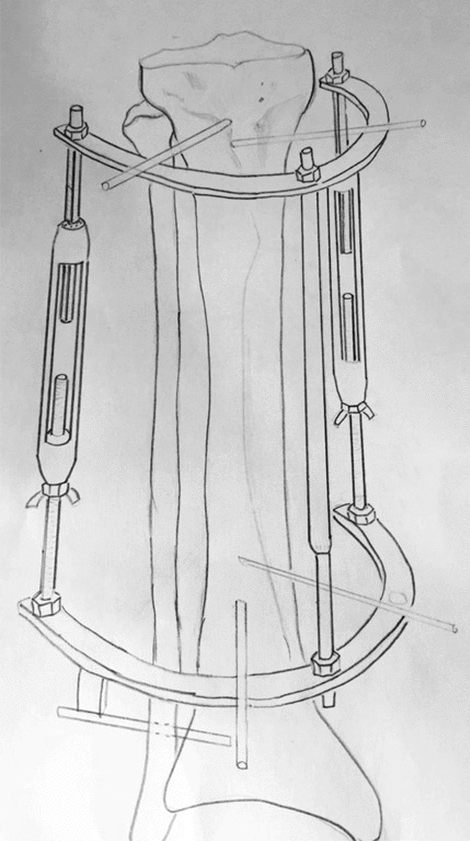
Fastening mechanism of advanced rod monolateral apparatus of external fixation on the patient’s lower leg.

For the experimental group, an external monolateral rod fixator based on the Sivash apparatus with advanced telescopic distraction rods and bars was applied (Figs. 1, 2). Elements of external supports of the device are quite small in size and have a shape of half rings. In terms of technical characteristics, these devices possess a resonance capacity and reliable in the fixation mechanism. Specifically, the distraction system consists of central, lateral, and medial telescopic blocks. The units have right-hand and left-hand threads with an adjustable rotating control mechanism.

### The procedure

Patients at the stage of preoperative preparation were mandatorily examined by a number of specialists, such as pediatricians, endocrinologists, neurologists, cardiologists, and otolaryngologists, for the purpose to identify accompanying pathology and prevent intra- and postoperative complications. Patients and parents were trained to take care of the device and the rods.

The operations in both groups were performed simultaneously on two similar segments according to the scheme lower leg-lower leg, upper arm-upper arm, and femur-femur.

A corticotomy in the experimental group was performed in the middle third of bone diaphysis. In the control group, an osteotomy with a chisel was performed in the lower third of the bone diaphysis, where the access was more favorable.

In the experimental group, the elaboration of movements in the adjacent joints began from the second day after the operation to prevent contractures. To avoid heel tendon tension, the foot support was set according to the scale of pain intensity. From the third day after surgery, the patient was verticalized with the help of walkers. The magnitude of the load depended on the pain syndrome, swelling of the distal parts of the limb, and the psychological condition of the patient. The elaboration of joints was not emphasized in the control group.

In both groups, distraction began from day 8 with an average daily step rate of 1 mm/day in 3 distraction planes by the method of 24-h limb lengthening in automatic Shevtsov-Popkov mode^20^. Control inspections with X-rays were performed every 10 days. Stability of external fixation mechanisms, the dermis status in the places of rods and spokes entry, the amount of movements in adjacent joints, bearing capacity of the limbs, and the presence of neurological or vascular disorders were estimated on the examination. Based on the radiological view of the regenerate and physical examination of the patient, a correction of the distraction rate, i.e., either reduction to 0.75 mm/day or increase to 2 mm/day, was performed. Regenerate distraction rate was decreased in the case of suspected knee or ankle joint contractures appearance due to heel tendon shortening (equinus). At the same time, intensive gymnastics and anti-inflammatory therapy were assigned. The decision for distraction acceleration was taken based on the results of X-ray analysis, as well as after estimating the condition of soft tissues and their extensibility. Upon reaching the planned possible segment length (8–10 cm in the experimental group), the distraction of the regenerate was terminated.

The quality of distraction was estimated under the mask anesthesia. The apparatus of external fixation was partially dismantled by easing the rods, and the regenerate stability was estimated under the control of the electron-optical converter in two planes in real-time. The evaluation of the mechanical stability of a regenerate was performed only in the experimental group, and for the control group, no data was available. At the satisfactory condition of the regenerate, the dynamic control was carried out by X-ray analysis in 2 projections for 7 days. If no pain or discomfort in the area of the regenerate was observed, the apparatuses were dismantled.

For both observed groups, the expected deviation in the process of bone elongation by external fixation mechanisms, i.e., the varus, valgus, and antecurvation deformity angles of the resulting bone regenerate, were evaluated and compared. Also, the incidence of infectious complications in the area of fixation spokes and rods placement was assessed during distraction. The level of pain was estimated according to the Wong-Baker faces pain rating scale on the 2nd and 18th days after surgery (10 days from the beginning of the distraction process).

Partial or absolute post-distraction shortening of the Achilles tendon was eliminated by needle aponeurotomy or achillotenotomy by Stayer in the experimental group, while for all patients in the control group, an open Z-achillotenotomy was applied.

When employing a circular multi-axis system, the data for the control group were obtained prior to the introduction of rod monolateral external fixation apparatus with an advanced controlled distraction system. For the wearing period of this system, some of the Dr. Paley performance criteria^21^, were not recorded since the surgery was performed by other specialists. Therefore, to perform the efficacy evaluation, only the criteria available in the retrospective review were selected.

### Statistical analysis

The qualitative features were encoded in numbers in order to be included in the calculations. Determination of the patients' distribution by the values of indicators was carried out using the Shapiro–Wilk criterion (W). At *p* < 0.05, the null hypothesis of normal distribution was rejected. Descriptive statistics were performed using generally accepted methods. To compare patients in both groups by frequency of distributing qualitative characteristics, the chi-square criterion was applied. At *p* < 0.05, the null hypothesis on the normality of distribution was rejected. Correlation analysis was performed to determine linear relationships in pairs of indicators. The analysis of correlations in pairs was carried out using the Spearman criterion. At *p* < 0.05, the null hypothesis on the absence of connection between indicators was rejected. Statistical calculations were carried out with the help of the SPSS program.

### Ethical approval

An interview with the parents on the purpose of the planned surgery was held in advance. Afterward, the consent to perform the surgery and publish obtained results without identification was signed. All methods were performed in accordance with the relevant guidelines and regulations. The parent and/or legal guardian for study participations gave their written informed consent. The study was reviewed and approved by the Ethical Committee of the NCJSC “Astana Medical University”.

### Informed consent

Informed consent was obtained from all subjects involved in the study.

## Data Availability

All data will be available on request.
